# Temporal trends in prevalence and years of life lived with disability for hearing loss in China from 1990 to 2021: an analysis of the global burden of disease study 2021

**DOI:** 10.3389/fpubh.2025.1538145

**Published:** 2025-03-04

**Authors:** Yuhuan Sun, Yang Yi, Geyao Huang, Shihao Jiang, Yuchen Zhou, Hongkun Chen, Dahui Wang

**Affiliations:** ^1^School of Public Health, Hangzhou Normal University, Hangzhou, China; ^2^Engineering Research Center of Mobile Health Management System, Ministry of Education, Hangzhou, China

**Keywords:** China, hearing loss, disease burden, epidemiological study, temporal trends

## Abstract

**Background:**

Hearing loss (HL) poses a serious threat to the health and quality of life of Chinese population. This study analyzes the burden of HL in China from 1990 to 2021 and projects future trends in next 15 years.

**Methods:**

Data derived from the Global Burden of Disease (GBD) 2021 study were utilized. The join-point regression model was employed to calculate the average annual percentage change (AAPC) in the prevalence and years of life lived with disability (YLDs) of HL. Age-period-cohort analysis was conducted to assess age, period and cohort effects. Decomposition analysis was performed to analyze the impacts of aging, population and epidemiological change. ARIMA model was utilized for forecasting the burden of HL from 2022 to 2036.

**Results:**

From 1990 to 2021, the number of prevalence and YLDs of HL in China rose by 125.06 and 135.13%, with an average annual percentage change of 0.19 and 0.28% for age-standardized rate (ASR) of prevalence and YLDs, respectively. Age-period-cohort analysis indicated that the risks associated with ASR of prevalence and YLDs for HL increased with age. The period effects on the ASRs of prevalence and YLDs were generally increasing (relative risk [RR] 0.98–1.06 and 0.96–1.05). Cohort effects on the risk also rising (RR 0.90–1.41 and 0.83–1.26). Aging growth accounted for the largest proportion of the increase of the number of prevalence and YLDs (68.62 and 66.39%, respectively). The prevalence and YLDs rates are expected to stabilize from 2022 to 2036, while the age-standardized prevalence rate remains above 20%. The number of people suffering from HL will reach 573.8 million, while the number of YLDs will reach 16 million.

**Conclusion:**

The prevalent cases of HL have risen dramatically in China over the past 32 years, which expected to continue to grow by 2036, additional interventions such as enhancing primary hearing care services and boosting screening rates for HL are essential to alleviate the burden of HL, especially in the older adult population.

## 1 Introduction

Hearing loss (HL) refers to a pathological reduction in auditory sensitivity and is often classified as an invisible disability. Globally, more than 1.5 billion people experience some degree of hearing impairment during their lifetime, with at least 430 million requiring intervention (1).

If left undiagnosed and untreated, HL can significantly impair an individual’s ability to work, engage in social interactions, and maintain psychological well-being, resulting in substantial medical resource utilization and a considerable social burden (2, 3). For individuals, auditory deprivation can diminish their quality of life and hinder access to spoken communication, potentially affecting language development in children and increasing the risk of cognitive decline and dementia in older adults (4–6). And people with HL also face higher rates of unemployment, lower educational achievements, and economic challenges (7). Moreover, HL can result in emotional consequences such as anxiety, depression, loneliness and isolation (8). Additionally, unaddressed HL poses a global economic burden exceeding $980 billion annually (9), encompassing healthcare expenses, educational impacts, productivity losses, and societal costs.

According to the 2015 study, HL ranked as the fourth leading cause of YLDs globally, while the 2019 study ranked third position (10, 11). Given the significant impacts of HL, there is an imperative and pressing need to implement effective measures against it. The World Health Organization (WHO) actively advocated for the implementation of a resolution aimed at preventing deafness and hearing impairment in 2018. This involved bringing together all relevant sectors within the field of otology and hearing healthcare to collaborate, and highlighting the significance of accessible hearing care services (12). The Chinese government places great importance on the hearing health of the older adult and children, incorporating hearing health into the national development and health planning, and implementing a number of assistance programs. Since the government issued the “National Action Plan for Disability Prevention (2016–2020)” in 2016 (13), services including cochlear implant surgery and rehabilitation training have been provided for children with hearing disabilities aged 0 to 6. At the end of 2021, the government emphasized the need to strengthen early hearing screening and intervention for children (14), setting a goal to achieve a newborn hearing screening rate of more than 90% by 2025. In 2022, relevant departments further issued the “Health Aging Plan for the 14th Five-Year Plan,” including hearing tests for the older adult in the basic public health services (15).

A number of current studies on the global burden of HL indicate a consistent upward trend in global prevalence and YLDs, with significant health inequalities across countries and regions (10, 16, 17). In addition to broader population-wide studies, Guo et al. (11) specifically examined children and adolescents and found that the HL burden has increased and remains high in this population, with the burden being higher in less economically developed countries or regions. Jiang et al. (18) study further attributes the increasing global burden of HL primarily to population growth and aging.

According to China’s seventh national census in 2020, 264.02 million individuals were aged 60 and above, constituting 18.70% of the total population (19). As a nation experiencing rapid population aging, China faces a very heavy burden of HL. Existing studies on HL’s disease burden in China, spanning from 1990 to 2019, have primarily focused on estimating and predicting future development trends, analyzing the burden of disease of different grades of HL, and finding that the prevalence of HL cases will increase significantly (20). Because of the serious burden of HL, the Chinese government has actively promoted its prevention and treatment through the establishment and continual enhancement of management, technical guidance, and service systems (21, 22). Nonetheless, a considerable number of individuals with HL fail to seek or receive appropriate hearing healthcare (23). Enhanced focus and resources must be directed toward tackling HL, acknowledging its status as an escalating public health concern that spans across all age demographics.

Although previous studies have estimated the disease burden of hearing loss in 2019 and predicted trends for the next 15 years, there is a lack of longitudinal research examining the trends in hearing loss burden from the perspectives of age, period, and cohort. Furthermore, the impact of aging, population growth, and epidemiological changes on the epidemiology of hearing loss has not been explored. Therefore, this study aims to describe the prevalence and YLDs trends of HL in China from 1990 to 2021, assessing the contributions of population growth, aging, population growth, and epidemiological change on these trends. It will also analyze the temporal variations in the prevalence and YLDs of HL over the specified period, examining age, period, and cohort effects. Furthermore, it seeks to forecast the HL burden in China for the next 15 years.

## 2 Materials and methods

### 2.1 Study data

The 2021 Global Burden of Disease (GBD) study used the latest data and enhanced methods to assess health loss from 371 diseases, injuries, and 88 risk factors across 204 countries. The GBD database serves as a key resource for epidemiological research, guiding public health strategies and resource allocation (17, 24).

For our research, we accessed global and country-specific data from the Global Health Data Exchange (GHDx) query tool (http://ghdx.healthdata.org/gbdresults-tool) to assess the burden of HL, encompassing the number and age-standardized rates (ASR) of prevalence and YLDs from 1990 to 2021, with parameters set to “Cause” as “Age-related and other hearing loss,” “Measure” as “Prevalence” and “YLDs,” “Metric” as “Number” and “Rate,” and “Location” as “China.” Additionally, to forecast the burden of HL from 2022 to 2036, we derived the projected population data for China from the United Nations’ World Population Prospects 2024 Revision, which provides data stratified by year, age, and sex (25). We also applied the World (WHO 2000–2025) Standard population to standardize prevalence and YLDs. The accuracy and demographic representativeness of this data have been confirmed by research on various diseases utilizing GBD data from China.

### 2.2 Definition of HL and health metrics in HL estimation

GBD defines HL as any level exceeding 20 dB, which is measured as the softest audible sound an individual can hear in their better ear, calculated by the pure-tone average of audiometric thresholds at 0.5 kHz, 1 kHz, 2 kHz, and 4 kHz (16).

The prevalence refers to the count of individuals who are currently affected by a certain disease or condition, and it can be presented as a simple number or as a percentage of the total population (with the total population considered as 100). Beyond prevalence, the GBD study employs a unique metric known as YLDs to assess the impact of non-fatal health issues. YLDs is a metric used to measure the impact of non-fatal health issues by calculating the number of years individuals live with disabilities due to diseases or injuries. Specifically, the estimation of YLDs begins with determining the prevalence of a specific disease within a certain population. Subsequently, each health state is assigned a disability weight, which reflects the severity of the health state, ranging from 0 (perfect health) to 1 (equivalent to death). These weights are derived from comprehensive multinational surveys that compare various health states in terms of their severity (26). By applying probit regression analysis to the aggregated data, the relative severity of each health state is translated into disability weight values. YLDs facilitate the quantification of health conditions’ burdens by considering both how common they are and how much they affect the quality of life due to their severity.

### 2.3 Data analysis

Previous research has provided a comprehensive explanation of the GBD 2021 study’s methodology (27). In our analysis, we focused on the prevalence and YLDs of HL, computing 95% uncertainty intervals (UIs) for each dataset. The rates were expressed per 100,000 population. We decomposed the raw number of cases and YLDs by age, population, and epidemiological changes to assess the degree to which population growth, aging, and epidemiological change have contributed to HL over the past 32 years. All tests were two tailed and *p* < 0.05 was considered statistically significant. Statistical analyses and graphical representations were conducted using R 4.3.3.

### 2.4 Join-point regression model

Join-point analysis was employed to estimate the trends in the prevalence and YLDs of HL from 1990 to 2021. As proposed by Kim (28), piecewise regression was applied to break down the longitudinal changes into distinct segments and determine those with statistical significance. The annual percent change (APC) was derived from the geometric mean of the various annual percent changes extracted from the regression analysis. The average annual percentage change (AAPC) serves as a composite measure of the trend over a defined period, calculated as a weighted average of the APC, which enables the description of the average APC over multiple years with a single value. The APC and AAPC were considered statistically significant if their 95% confidence interval did not include the null hypothesis of zero change, with a *p*-value less than 0.05. The analysis was executed using the Join-point Regression Program, Version 5.1.0.

### 2.5 Age-period-cohort model

The age-period-cohort model is a tool used to analyze the impact of age, period, and cohort on health outcomes. The age effect captures the varying risks of outcomes across different age groups. The period effect characterizes the risk of specific health outcomes at various times. The cohort effect reflects the fact that groups belonging to each same birth cohort have different outcomes due to altered birth patterns or different health risk exposures. The intrinsic estimator method integrated into the age-period-cohort model was employed to get the net effects for three dimensions (29).

To implement the age-period-cohort model, the dataset was segmented into successive 5-year periods, spanning the period from 1992 to 2021. Data from 1990 and 1991 were excluded as they did not form a complete 5-year period. Furthermore, 20 age groups with 5-year intervals, and 25 cohorts were summarized. The mean level of age, period and cohort was selected as the reference groups (30). The relative risk (RR) values for each age, period and cohort were expressed as the independent risks relative to the reference group.

### 2.6 Autoregressive integrated moving average (ARIMA) model

The ARIMA model was utilized to analyze the time series data of ASR for prevalence and YLDs of HL from 1990 to 2021 and to forecast these values forward to 2036. The model requires the time series to be a stationary and stochastic sequence with a mean of zero, which is a combination of an autoregressive (AR) model and a moving average (MA) model (31). In the modeling process, the difference method is firstly employed for the purpose of stabilizing the time series data. The *auto.arima()* function is used to identify the most suitable model, optimized based on Akaike Information Criterion (32).

## 3 Results

### 3.1 HL in China

The case number of HL in China increased from 197,913,077(95%UI 187,282,240- 210,803,812) in 1990 to 445,413,952(95%UI 421,771,524- 471,934,850) in 2021, an increase of 125.06%. In addition, the age-standardized prevalence rate (ASPR) for HL was 20719.03 (95%UI 19,614.53- 22,041.30) in 1990, 22004.91 (95%UI 20895.30–23245.93) in 2021, and 0.19% (95% CI 0.18–0.21) in AAPC. The number of HL YLDs in China increased from 5,274,808(95%UI 3,572,950- 7,403,771) in 1990 to 12,402,438(95%UI 8,568,296- 17,253,150) in 2021, a rise of 135.13%. And the ASR of YLDs for HL was 577.47 (95%UI 394.72–803.41) in 1990, 630.67 (95%UI 433.51–874.80) in 2021, and 0.28% (95% CI 0.26–0.31) in AAPC. In both 1990 and 2021, the number and ASR of YLDs for HL were slightly higher in males than in females (Table 1 and Supplementary Table S1).

**Table 1:** The number and ASR of prevalence and YLDs of hearing loss in China from 1990 and 2021, and its temporal trends from 1990 to 2021.

Sex	Prevalence					YLDs (Years of life lived with disability)				
	1990		2021		1990–2021 AAPC (%- 95CI)	1990		2021		1990–2021 AAPC (%- 95CI)
	Number (95% UI)	ASR (95% UI)	Number (95% UI)	ASR (95% UI)		Number (95% UI)	ASR (95% UI)	Number (95% UI)	ASR (95% UI)	
Male	105,086,705 (99046121–112,394,775)	21608.14 (20436.66–23090.22)	228,010,463 (215482944–242,123,040)	22974.09 (21836.91–24302.43)	0.20 (0.18–0.21)	2,787,755 (1883539–3,936,677)	606.69 (414.57–847.76)	6,358,410 (4369217–8,879,812)	667.54 (457.15–926.96)	0.31 (0.28–0.34)
Female	92,826,372 (87872343–98,902,192)	19765.45 (18736.43–21018.71)	217,403,490 (205789344–231,097,202)	20981.02 (19941.28–22191.93)	0.19 (0.18–0.20)	2,487,053 (1693821–3,474,669)	546.87 (374.08–759.71)	6,044,028 (4181621–8,359,144)	592.27 (409.12–818.94)	0.26 (0.24–0.28)
Both	197,913,077 (187282240–210,803,812)	20719.03 (19614.53–22041.30)	445,413,952 (421771524–471,934,850)	22004.91 (20895.3–23245.93)	0.19 (0.18–0.21)	5,274,808 (3572950–7,403,771)	577.47 (394.72–803.41)	12,402,438 (8568296–17,253,150)	630.67 (433.51–874.80)	0.28 (0.26–0.31)

ASR, age-standardized rate (per100,000 population); AAPC, average annual percentage change.

**Figure 1:**
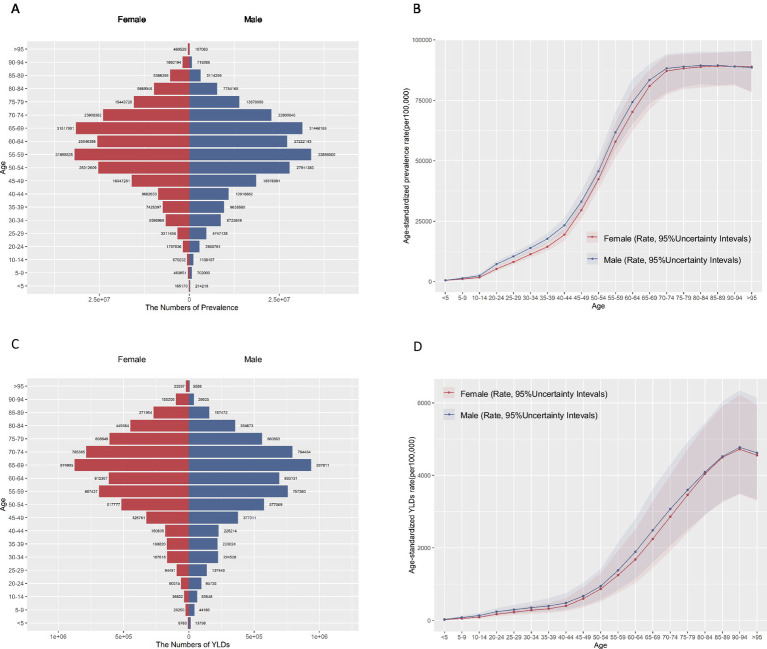
Age-specific numbers and age-standardized rates of prevalence and YLDs of hearing loss in China, 2021. **(A)** Age-specific prevalence number. **(B)** Age-standardized prevalence rate. **(C)** Age-specific YLDs number. **(D)** Age-standardized YLDs rate. YLDs, years of life lived with disability.

HL was predominantly prevalent among individuals aged over 45 years old, increasing notably between the ages of 40 and 69. The ASPR reached its highest point at 85–89 years in both females (89.14, 95%UI 80.79–94.67) and males (89.47, 95%UI 81.25–95.12) (Figures 1A,B and Supplementary Table S1). Similarly, YLDs showed a substantial increase after age 40, with the highest ASR of YLDs also peaking at 90–94 years (Figures 1C,D). It is noteworthy that the male population exhibited higher ASR of prevalence and YLDs of HL than the female population. Over the years 1990 to 2021, both sexes saw gradual increases in the number of prevalence and YLDs, with slight fluctuations in sex-specific ASR (Supplementary Figure S1). Overall the burden of disease has been consistently lower for females than for males.

### 3.2 Decomposition analysis of prevalence and YLDs

**Figure 2:**
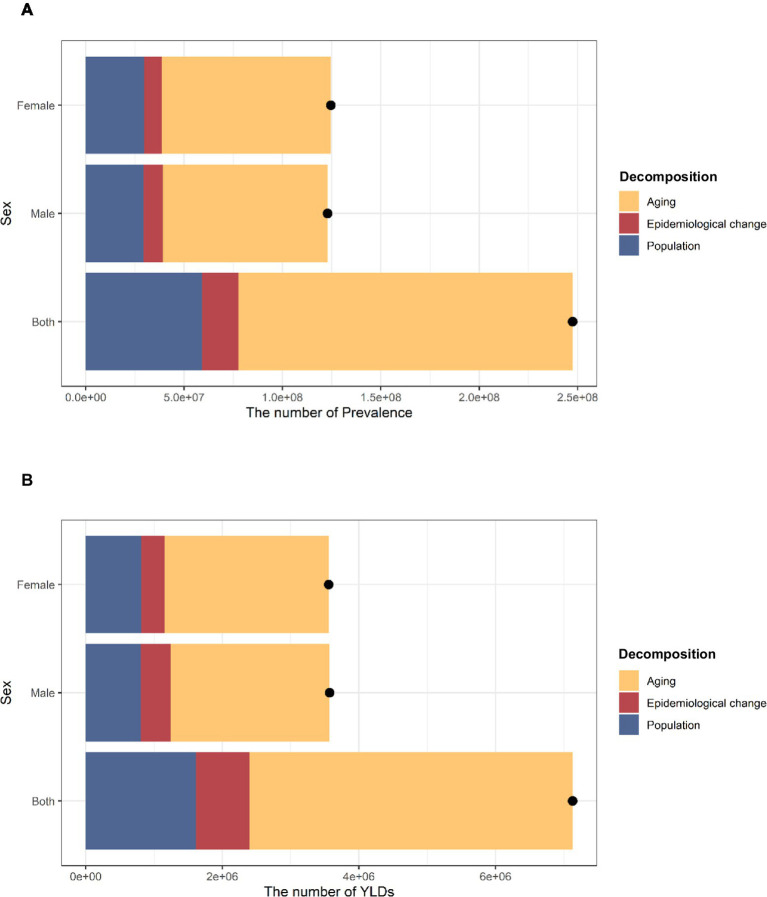
Changes in prevalence and YLDs of hearing loss in China according to population-level determinants of population growth, aging, and epidemiological change from 1990 to 2021.The black dot represents the overall value of change contributed by all three components. The positive value shown for each component indicates a corresponding increase in HL prevalence and YLD attributed. **(A)** Changes in prevalence. **(B)** Changes in YLDs. YLDs, years of life lived with disability. HL, hearing loss.

There has been a notable rise in the number of prevalence and YLD in China over the past 32 years. In the total population, the increase in disease burden in terms of prevalence was mainly attributable to aging (68.62%) and population growth (23.86%). Similarly, the main reasons for the increase in the number of YLDs are aging (66.39%) and population growth (22.64%). Male or female groups showed similar results (Figure 2 and Supplementary Table S2).

### 3.3 Join-point analysis of ASRs of prevalence and YLDs of HL

**Figure 3:**
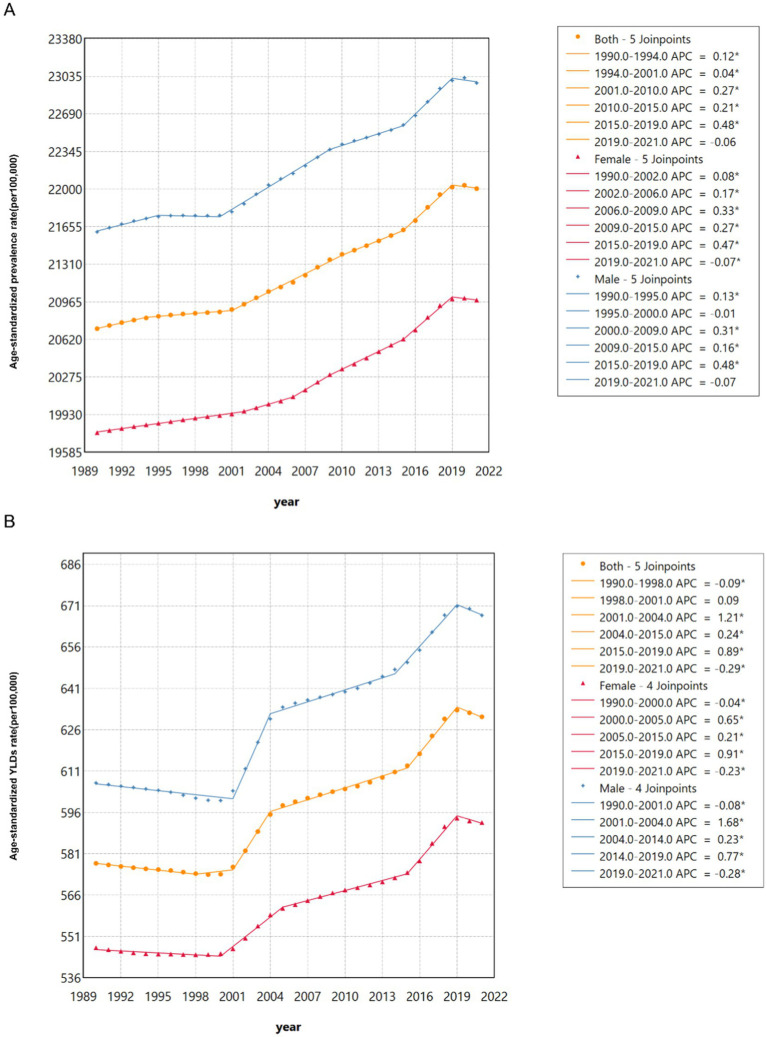
Join-point regression analysis of the sex-specific age-standardized prevalence rate for hearing loss in China from 1990 to 2021. **(A)** Age-standardized prevalence rate. **(B)** Age-standardized YLDs rate.

Join-point regression analyses of the ASPR for HL in China from 1990 to 2021 are shown in Figure 3A. The results indicated a significant increase in ASPR of HL from 2015 to 2019 in both the male (APC = 0.48, 95% CI: 0.42–0.54) and female (APC = 0.47, 95% CI: 0.44–0.50) populations. However, a decreasing trend was observed in females from 2019 to 2021 (APC = - 0.07, 95% CI: −0.13- -0.01). Since 2005, the male population (APC =0.23 (2004–2014), 95% CI: 0.21–0.25; APC =0.77(2014–2019), 95% CI: 0.70–0.84) and the female population (APC = 0.65 (2005–2015), 95% CI: 0.59–0.70; APC =0.21(2015–2019), 95% CI: 0.20–0.23; APC =0.91(2019–2021), 95% CI: 0.82–1.00) had a significant increasing trend in ASR of YLDs. However, the ASR of YLDs exhibited a decline for males from 2019 to 2021 (APC = −0.28, 95% CI: −0.50- -0.06) and for females from 2000 to 2005(APC = −0.04, 95% CI: −0.06- -0.03) (Figure 3B). It is noteworthy that the females exhibited a lower AAPC of ASR of YLDs compared to males (Supplementary Table S3).

### 3.4 Age, period and cohort effects on ASRs of prevalence and YLDs of HL

**Figure 4:**
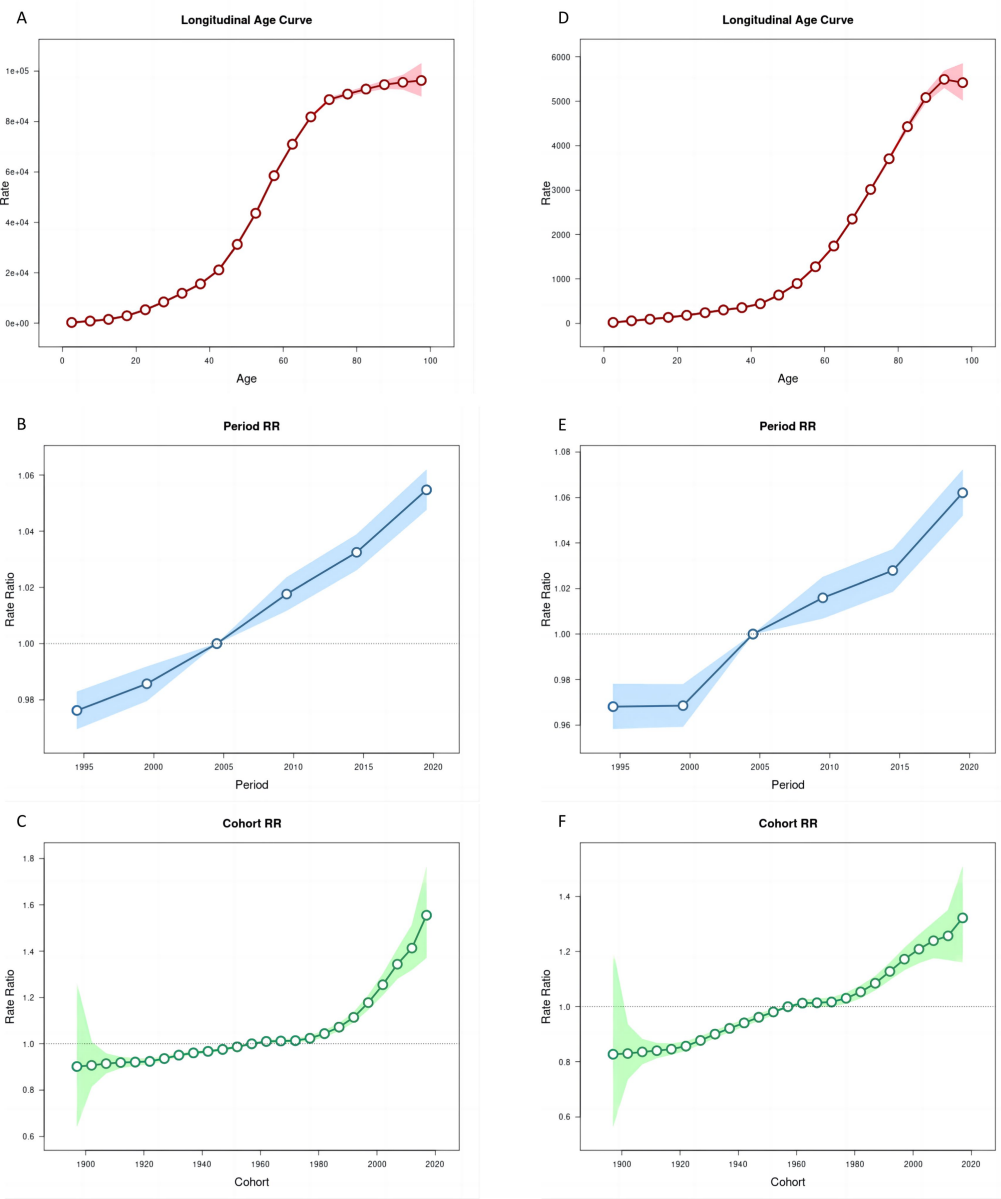
Age, period and cohort effects on age-standardized rates of prevalence and YLDs of hearing loss in China during 1990–2021. **(A,D)** Longitudinal age curves. **(B,E)** Period effects. **(C,F)** Cohort effects, the shaded regions represent their 95% CIs. YLDs, years lived with disability.

**Figure 5:**
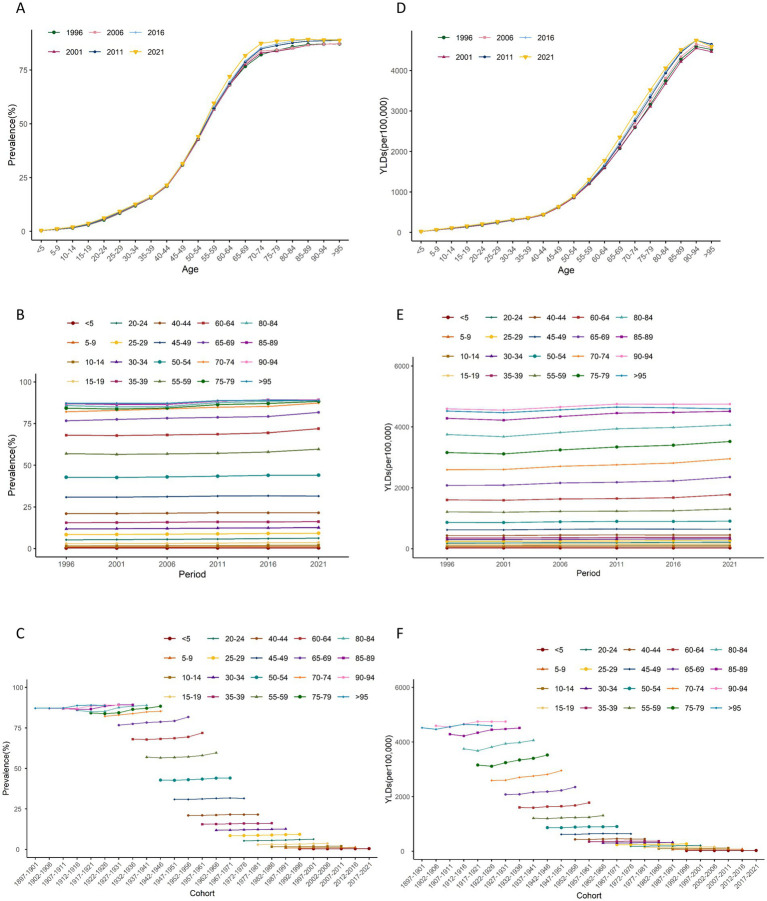
Trends of age-specific, period-based and cohort-based variation of age-standardized rates of prevalence and YLDs of hearing loss in China. **(A,D)** Age-specific age-standardized rates of prevalence and YLDs of hearing loss; **(B,E)** period-based age-standardized rates of prevalence and YLDs of hearing loss; **(C,F)** cohort-based age-standardized rates of prevalence and YLDs of hearing loss. YLDs, years lived with disability.

Age-period-cohort analyses help us to understand differences in health risks faced by different birth cohorts at different ages or periods. Age effect on the ASR of prevalence and YLDs of HL showed an increasing trend (Figures 4A,D, 5A,D).

The period effect on the ASR of prevalence and YLDs of HL increased continuously (Figures 4B,E, 5B,E). The trends in ASPR remained relatively stable among younger groups but exhibited an increasing pattern across the 55–59 age group over time. From 1996 to 2021, the relative risk (RR) of ASR of prevalence and YLDs rosed from 0.98 (95% CI 0.97–0.98) to 1.06 (95% CI 1.05–1.06), and from 0.97 (95% CI 0.96–0.98) to 1.06 (95% CI 1.05–1.07) in 2021, respectively (Supplementary Table S4).

The cohort effect showed that among earlier birth cohorts was lower [RR cohort (1987–1901) = 0.90, 95% CI 0.65–1.26] but has risen in more recent cohorts [RR cohort (2017–2021) = 1.41, 95% CI 1.32–1.51]. Similarly, the cohort risk of YLDs was lower in earlier birth cohort [RR cohort (1987–1901) = 0.83, 95% CI 0.57–1.21] and increased among recent cohorts [RR cohort (2017–2021) = 1.26, 95% CI 1.17–1.35] (Figures 4C,F, 5C,F).

### 3.5 Prediction of HL prevalence and YLDs in China by 2036

**Figure 6:**
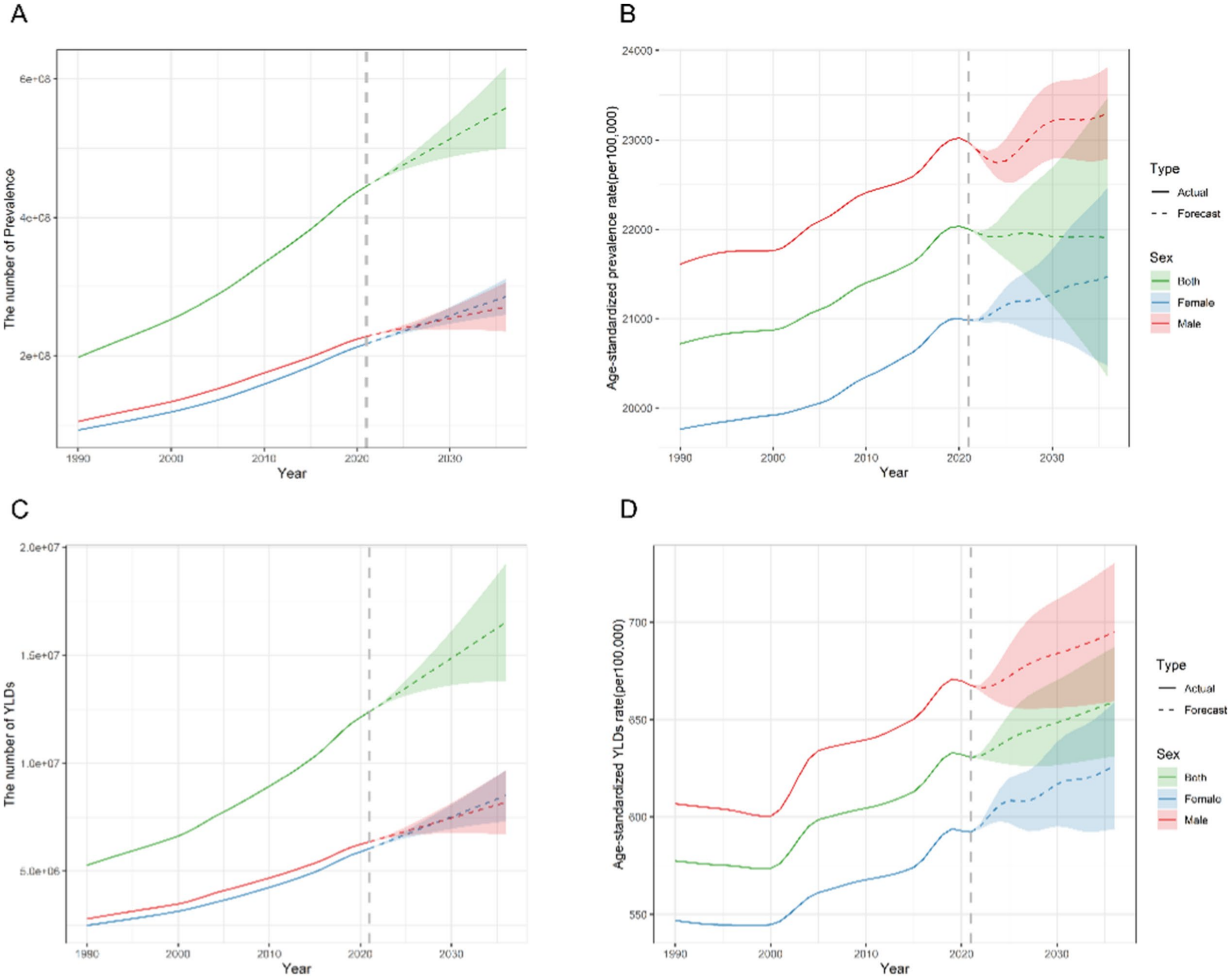
Predicted trends of the number and age-standardized rate of prevalence and YLDs of hearing loss in China over the next 15 years (2022–2036). The solid and dashed lines represent the true trend in hearing loss from 1990 to 2021; the shaded areas represent the projected trend and its 95% CI. **(A,C)** Predicted trends of prevalence. **(B,D)** Predicted trends of YLDs. YLDs, years lived with disability.

The ARIMA model was employed to forecast the trajectories in HL prevalence and YLDs over the next 15 years. All models have undergone rigorous diagnostic checks, including stationarity tests and white noise tests, and the results indicate satisfactory model fit (Supplementary Tables S6, S7). According to the projections, overall HL prevalence is expected to experience a slight decline. The ASPR is expected to stabilize at 21.91% by 2036, a minor change from the 22.00% recorded in 2021 (Figure 6). There are slight differences by sex, with the ASPR reaching 23.30 and 21.47% for males and females, respectively, in 2036 (Supplementary Table S5).

Future trends indicate that the ASR of YLDs would remain at the level of 659.24 per 100,000 in 2036 (compared with 630.67 in 2021) (Figure 6). And the ASR of YLDs for females is predicted to increase from 595.34 in 2022 to 626.17 in 2036 (per 100,000). Over the same period, the ASR of YLDs for males is predicted to rise from 666.31 in 2022 to 694.86 in 2036 (per 100,000) (Supplementary Table S5).

## 4 Discussion

In 2021, China accounted for 28.8% of the global population with HL. Monitoring temporal trends in the burden of HL is essential for identifying emerging challenges and adapting healthcare systems accordingly. Approximately 445.4 million individuals in China, representing over 30% of the total population, were affected by HL, with an ASPR of 22.00%. This exceeds the global average of 18.07%, as well as the ASR in the United States (13.73%) and India (19.21%) (33). The “China Hearing Health Report (2021)” points out that presbycusis accounts for the leading cause of hearing disability in China, at 51.61% (34). Moreover, according to the seventh national population census in China in 2020, China remains the most populous country in the world (35), with the proportion of the population aged 60 and above reaching 18.70%, and is also one of the fastest-aging populations globally (36). Hearing loss is highly correlated with age, and the risk of developing hearing loss significantly increases as one gets older. Therefore these factors contribute to China’s higher ASPR. Globally, hearing loss accounted for over 44.4 million YLDs, ranking as the fourth leading cause of YLDs in the GBD study. Similarly, in China, hearing loss was responsible for more than 12.4 million YLDs, making it the leading cause. Furthermore, in 2021, the ASR of YLDs due to hearing loss in China was higher than the global average, at 630.67 per 100,000 population compared to 525.87 per 100,000 globally. For the past 32 years, despite being a non-life-threatening condition, HL showed a high prevalence and YLDs but has not garnered the attention it warrants (16). Prejudices about HL often prevent timely treatment (37, 38). Untreated HL can significantly affect work performance, interpersonal interactions, mental health, and quality of life, which must be acknowledged and addressed with seriousness (7–9, 39).

Between 1990 and 2021, there was a notable rise in the number of prevalence and YLDs with an increase of 125.06 and 135.13%, while the ASR of prevalence and YLDs increased slowly with an AAPC of 0.19 and 0.28%, respectively. With the aging and population growth, more individuals are likely to experience hearing impairments, leading to an increase in the number of YLDs. However, the implementation of health measures, advancements in medical technology, and the prevention and control strategies for diseases may to some extent offset the impact of population growth and aging on the disease burden of HL, resulting in a slow increase in ASR. And analysis of age-period-cohort effects highlighted distinct patterns in the ASRs of prevalence and YLDs, primarily driven by variations in age, period, and cohort factors. The prevalence cases of HL notably increased with age, with an accelerated increase in ASPR from the age of 45 years onwards. In individuals aged 60 and above, the ASPR of HL ranged from 72.3 to 89.3% (Supplementary Table S1), which was higher than the findings of the GBD Study 2019 (20). Specifically, a significant upward trend in the ASPR was detected for the 55–59, 60–64 and 65–69 age groups from 2017 to 2021 (Figure 5) (20). Previous studies have linked hearing impairment to cognitive decline, depression, anxiety, frailty, and difficulties in performing activities of daily living (ADLs) (40, 41). Consequently, there is an urgent need for scientific diagnosis, management, and rehabilitation of older individuals with HL. However, widespread HL screening in China is hindered by the high cost of specialized equipment, and a shortage of trained technicians (42–44). Although evidence suggests that hearing aids can help mitigate cognitive decline by improving daily auditory experiences, their adoption in China remains limited due to cultural factors and health-related beliefs (23). Hearing aid producers and distributors make an estimation that hearing aid production currently meets less than 10% of global need, a percentage that is even lower (3%) in low-and middle-income countries (45).

Additionally, approximately 3.32 million children aged 14 and younger in China continue to experience varying degrees of HL. This represents a significant decline in both the prevalence of HL cases and the ASPR compared to 2019, and the ASPR is now lower than the global rate recorded in 2021 (11, 20). This improvement is likely attributed to the widespread implementation of universal newborn hearing screening, along with timely diagnosis, intervention, and rehabilitation services. Research has demonstrated that children in regions with early newborn hearing screening achieve better developmental outcomes, including enhanced social and motor skills, as well as improved overall quality of life, compared to those in areas with delayed screening (46, 47). In China, the coverage of national newborn hearing screening has increased substantially, rising from 29.9% in 2008 to 86.5% in 2016, and surpassing 90% by 2022 (46, 48). Despite this progress, hearing loss in children remains a critical issue, as it can negatively impact speech development, reading abilities, and cognitive function, ultimately hindering educational progress and social integration (49). Furthermore, otitis media and meningitis, as the two causative factors that lead to HL in children, are preventable (50). It is essential to continue improving the diagnosis and treatment of HL in children.

Period effects encompass the changes in medical advancements, diagnostic methods, economy, and culture that influence the burden of HL within a specific time frame. From 1992 to 2021, the RR of ASR of prevalence and YLDs of HL escalated from 0.98 to 1.06 and from 0.97 to 1.06, respectively, which aligns with societal progress. With ASR remaining stable, the rising prevalence and YLDs associated with HL in China are primarily driven by aging (68.62%) and population growth (23.86%), in line with the results from the GBD 2019 Hearing Loss Collaborators (16) and previous studies performed by Wang et al. (20). Despite efforts to improve early detection, diagnosis, and treatment of HL, these interventions have not been sufficient to offset the effects of aging and population growth. While global trends suggest that population growth (60.84%) and aging (35.35%) are the main drivers of increased HL prevalence and YLDs, as noted by Jiang et al. in 2019 (18), the situation in China is distinct. In China, aging is the predominant factor contributing to the rising burden of HL, indicating that the challenge posed by an aging population is more pronounced compared to global trends. This finding aligns with existing research on the global epidemiology of hearing loss (51–53).

Cohort effects emphasize socioeconomic, behavioral and environmental exposures and risks are linked to early life experiences across various birth cohorts. Analyses of birth cohort data from different age groups suggest that the risk of HL prevalence and YLDs increased in early birth cohorts, whereas the risk of HL remained stable in recent birth cohorts. This trend could be attributed to improvements in diagnostic accuracy and screening methods, heightened public and professional awareness, and changes in environmental and social determinants. HL arises from a mix of genetic, health, lifestyle, and environmental influences (54). Preventive measures can either forestall the development of HL or decelerate its progression across all age groups. In clinical practice, it is essential to exercise caution when administering ototoxic medications, broaden the scope of screening, ensure accurate diagnosis of patients with HL, initiate prompt treatment, and provide appropriate hearing aids when necessary (16, 55).

By 2036, it is anticipated that the population in China with HL will continue to grow, potentially reaching 573.8 million individuals, which would account for over 40% of the total population, with the ASPR of HL remaining relatively stable at approximately 22%. Moreover, HL will be responsible for over 16.5 million YLDs in China, highlighting the significant challenges that the country will continue to face in addressing the increasing burden of HL. In this study, we noted that males exhibited a higher burden of HL compared to females in terms of both prevalence and YLDs, a finding that aligns with earlier studies (20, 56). It is significant to observe that the females exhibited a lower AAPC of ASR of YLDs compared to males, which suggests that ASR of YLDs of HL for male increases at a faster average annual rate than for females. And within the next 15 years, the ASR of the prevalence and YLDs are likely to remain higher in males. This disparity could be attributed to variations in hormone production between sexes (57), as well as differences in occupational exposures and lifestyles (58). Estrogen is the major female hormone involved in reproductive functions, which could modulate cochlear physiology (59). And compared to female, male may be more frequently exposed to high-noise environments due to occupational reasons, such as working on construction sites or in factories. This constant exposure, along with habits like smoking and drinking, and various chronic diseases, significantly increase their risk of hearing loss. Additionally, female may place greater emphasis on hearing protection, taking more preventive measures to reduce the risk of hearing damage, such as avoiding prolonged stays in environments with high noise levels or correctly using protective equipment like earplugs when necessary.

To better address the significant burden of severe hearing loss, the WHO and governments around the world are actively taking measures. An objective of Healthy People 2030 in U.S. is to increase the proportion of adults with hearing loss who use hearing aids from 24.4% in 2018 to 26.4%. The U.S. Congress has proposed legislation that allows patients to access audiologists directly and simplifies Medicare coverage, with an estimated saving of $108 million over a decade (60). In addition to actively carrying out neonatal hearing screening, India has also vigorously conducted school hearing screening. Regular school-based health check-ups and training of teachers, social workers and school nurses to identify hearing disability can help in early detection of ear pathologies (61). China, as a country bearing a heavy burden of hearing loss, is expected to see this burden continue to grow, especially among the older adult population. The key to tertiary prevention of HL is hearing screening, which has been effective in the newborn population and needs to be extended to other groups, especially the older adult. It is imperative for China to embrace the “HEARING” intervention strategies promoted by the WHO. This comprehensive program targets individuals of all ages with HL and includes initiatives such as hearing assessments, prompt treatment for ear conditions, provision of assistive devices like hearing aids, functional restoration for those with HL, and noise risk mitigation (1). Since 2015, the National Health Commission has established a national technical guidance group for deafness prevention and treatment, dedicated to the early detection, diagnosis, and treatment of ear diseases (62). State Council documents and plans propose subsidies for the allocation of assistive devices in urban and rural areas, and include hearing diagnosis and treatment in the “Healthy China 2030” Plan Outline (15, 63). The National Health Commission has strengthened the construction of medical service capabilities, standardized clinical diagnosis and treatment as well as drug use, promoted technologies such as cochlear implantation, improved the rehabilitation system, and provided family doctor contract services. From 2024 to 2027 (64), the National Health Commission will carry out a hearing health promotion campaign for the older adult to raise public awareness and support for early intervention. The Ministry of Education has also included related majors in the higher education system, with more than 190 colleges and universities now offering these majors, alleviating the shortage of rehabilitation personnel (65). Through multifaceted efforts, including policy support, public education, advances in medical technology, and early intervention, the challenges of HL in China and Globe burden HL will be reduced effectively.

## 5 Strengths and limitations

This study systematically and comprehensively analyses the prevalence and burden in terms of YLDs of HL in the general population of China in 2021, as well as changes in the burden over the past32 years. The report also provides projections of the disease burden over the next 15 years. This latest research holds significant positive implications for both China and the international community. It not only equips the Chinese government with the most up-to-date data needed to formulate effective policies to address health challenges related to HL, but also serves as a valuable reference for other nations encountering analogous aging issues and similar regions lacking adequate hearing health services.

It is crucial to acknowledge its limitations when interpreting and utilizing the results. Firstly, the data relied heavily on the accuracy and completeness of surveillance systems and registries. Incomplete reporting or underdiagnosis of HL cases, especially in rural areas or among older populations, may result in an underestimation of the true burden of HL. Secondly, the research’s dependence on the GBD 2021 database inherits the same limitations, including potential biases in data collection and the reliance on modeling techniques to address missing or incomplete data. The GBD’s estimates come with uncertainty intervals that may not be fully captured in the study’s findings. Thirdly, while link-point regression models, age-period-cohort analyses and ARIMA models are reliable methods for analyzing trends and making projections, their accuracy hinges on the quality and consistency of the input data; any inaccuracies or inconsistencies can skew model predictions. Fourthly, the study’s predictive models did not account for potential future shifts in economic conditions, public health policies, or environmental factors that could significantly influence the HL burden. Future changes in economic conditions, such as economic growth and increased public health budgets, along with policy changes, including legislation for hearing health care and enhanced preventive measures, are expected to significantly reduce the prevalence and burden of HL. In addition, rising environmental noise levels increase the risk of HL. Finally, the study notes that looking at national data alone might hide regional differences in hearing loss (HL) in China. Differences in healthcare, wealth, and attitudes toward HL can lead to varying burdens across regions. Urban–rural development disparities and lower medical care in poor, remote areas make it hard for vulnerable groups to get ear and hearing services, increasing HL. Wealthier families can provide better care and rehab for their kids, reducing HL in children (34). Also, some regions do not focus enough on HL, which can lead to more cases and a heavier disease burden.

## 6 Conclusion

HL is a critical public health concern in China. The burden of HL has grown rapidly alongside the aging of the population over the past 32 years, and this trend is projected to continue through 2036. Given the ongoing concern regarding the future burden of HL, it is imperative for the government to enhance the development of hearing health services, adopt a tiered prevention strategy for HL, broaden the reach of HL screening, and ensure timely identification for the implementation of cost-effective intervention and treatment programs, especially for the older adult population.

## Data Availability

The original contributions presented in the study are included in the article/Supplementary material, further inquiries can be directed to the corresponding author.
