# Supplementary material for: Temporal trends in prevalence and years of life lived with disability for hearing loss in China from 1990 to 2021: an analysis of the global burden of disease study 2021
Source: Front Public Health. 2025 Mar 4;13:1538145. doi: 10.3389/fpubh.2025.1538145 (PMC11913668; doi:10.3389/fpubh.2025.1538145)
Supplement: Supplementary file 1 [file Supplementary_file_1.docx]

Supplementary Material

# Supplementary Figures and Tables

## Supplementary Figures

**Supplementary Figure 1.** Trends in the all-age cases and age-standardized rates of prevalence and YLDs of hearing loss by sex from 1990 to 2021. (A) Number and age-standardized rate of prevalence; (B) Number and age-standardized rate of YLDs; YLDs, years lived with disability.

## Supplementary Tables

**Supplementary Table1**. The number and ASR of prevalence and YLDs of hearing loss in China in 2021 by age and sex.

| Sex | Age | Prevalence | | YLDs (Years of life lived with disability) | |
| --- | --- | --- | --- | --- | --- |
|  |  | Number | Rate(95%UI) | Number | Rate(95%UI) |
| Both | <5 | 379388 | 488.5 (373.0- 621.6) | 22560 | 29.0 (17.3- 45.8) |
|  | 5-9 | 1165851 | 1217.3 (847.9- 1646.8) | 70430 | 73.5 (44.1- 111.4) |
|  | 10-14 | 1778399 | 2063.3 (1502.3- 2713.7) | 100770 | 116.9 (71.9- 178.5) |
|  | 15-19 | 2764967 | 3702.8 (3005.0- 4583.2) | 121202 | 162.3 (103.6- 236.6) |
|  | 20-24 | 4606598 | 6295.4 (5225.8- 7476.4) | 156050 | 213.3 (139.6- 301.4) |
|  | 25-29 | 8058588 | 9318.2 (8207.4- 10541.1) | 231931 | 268.2 (171.6- 386.6) |
|  | 30-34 | 15310819 | 12637.6 (11052.2- 14282.6) | 392126 | 323.7 (208.3- 462.7) |
|  | 35-39 | 17060957 | 16100.8 (14417.4- 18174.9) | 388844 | 367.0 (233.4- 548.3) |
|  | 40-44 | 19581515 | 21392.7 (18911.3- 24183.7) | 409049 | 446.9 (279.0- 686.3) |
|  | 45-49 | 34624272 | 31384.9 (27821.4- 35374.2) | 702773 | 637.0 (387.8- 981.4) |
|  | 50-54 | 53223992 | 44038.1 (39466.7- 49585.6) | 1094846 | 905.9 (545.9- 1359.8) |
|  | 55-59 | 65797328 | 59847.1 (52853.1- 68407.2) | 1444797 | 1314.1 (800.5- 2037.7) |
|  | 60-64 | 52768532 | 72280.5 (63740.5- 81492.4) | 1305438 | 1788.1 (1091.5- 2638.9) |
|  | 65-69 | 62966154 | 82090.3 (72670.1- 88704.4) | 1812805 | 2363.4 (1522.9- 3480.5) |
|  | 70-74 | 46709028 | 87640.0 (78060.7- 93750.1) | 1579819 | 2964.2 (1987.1- 4138.6) |
|  | 75-79 | 29313785 | 88510.6 (79963.7- 94168.3) | 1167542 | 3525.3 (2451.2- 4822.0) |
|  | 80-84 | 17624112 | 89047.3 (81120.4- 94842.8) | 804037 | 4062.5 (2933.1- 5411.5) |
|  | 85-89 | 8502618 | 89259.3 (80816.3- 94770.0) | 429426 | 4508.1 (3269.3- 5947.0) |
|  | 90-94 | 2609460 | 88999.7 (81258.5- 94981.8) | 138811 | 4734.4 (3484.8- 6252.3) |
|  | 95+ | 567592 | 88811.3 (78559.3- 95189.9) | 29185 | 4566.6 (3314.8- 5985.1) |
| Female | <5 | 165170 | 458.3 (344.2- 582.9) | 8763 | 24.3 (14.7- 38.2) |
|  | 5-9 | 463851 | 1034.8 (718.2- 1420.5) | 26250 | 58.6 (35.1- 89.5) |
|  | 10-14 | 670232 | 1667.2 (1196.3- 2244.6) | 36822 | 91.6 (56.6- 142.1) |
|  | 15-19 | 1035717 | 2994.3 (2391.3-3750.1) | 44684 | 129.2 (82.7- 188.6) |
|  | 20-24 | 1797836 | 5240.5 (4270.3- 6374.3) | 60315 | 175.8 (114.2- 255.3) |
|  | 25-29 | 3311450 | 8103.1 (7081.5- 9316.6) | 94491 | 231.2 (149.9- 333.0) |
|  | 30-34 | 6586969 | 11265.7 (9854.7- 12958.9) | 167618 | 286.7 (182.5- 412.9) |
|  | 35-39 | 7425397 | 14391.1 (12738.4- 16299.3) | 168820 | 327.2 (208.4- 485.0) |
|  | 40-44 | 8662633 | 19419.3 (16859.7- 21832.5) | 180835 | 405.4 (255.6- 615.3) |
|  | 45-49 | 16047281 | 29574.8 (26094.8- 33431.0) | 325761 | 600.4 (366.2- 917.2) |
|  | 50-54 | 25312609 | 42386.9 (38209.1- 47769.6) | 517777 | 867.0 (523.5- 1301.9) |
|  | 55-59 | 31898828 | 57935.9 (51307.7- 65815.6) | 687437 | 1248.6 (757.7- 1934.2) |
|  | 60-64 | 25546388 | 70221.3 (62363.7- 79400.3) | 612307 | 1683.1 (1024.5- 2508.9) |
|  | 65-69 | 31517991 | 80891.2 (71288.9- 87652.5) | 874995 | 2245.7 (1443.1- 3282.7) |
|  | 70-74 | 23908382 | 87129.8 (77483.1- 93442.8) | 785385 | 2862.2 (1916.6- 4005.2) |
|  | 75-79 | 15443728 | 88166.7 (79612.1- 94022.6) | 606649 | 3463.3 (2415.7- 4724.1) |
|  | 80-84 | 9869946 | 88783.4 (80193.9- 94383.4) | 449364 | 4042.2 (2913.0- 5361.1) |
|  | 85-89 | 5388359 | 89139.9 (80793.7- 94670.0) | 271954 | 4498.9 (3249.1- 5908.1) |
|  | 90-94 | 1890194 | 89027.7 (81036.5- 94983.4) | 100206 | 4719.7 (3488.3- 6216.8) |
|  | 95+ | 460528 | 88878.5 (78506.1- 95361.1) | 23597 | 4554.0 (3297.4- 5961.7) |
| Male | <5 | 214218 | 514.6(388.5-662.3) | 13798 | 33.2(19.7-52.2) |
|  | 5-9 | 702000 | 1378.0 (964.6- 1854.6) | 44180 | 86.7 (51.9- 132.1) |
|  | 10-14 | 1108167 | 2409.5 (1772.5- 3175.2) | 63948 | 139.0 (85.1- 212.2) |
|  | 15-19 | 1729250 | 4314.2(3509.6-5343.3) | 76518 | 190.0(121.4-277.2) |
|  | 20-24 | 2808761 | 7226.5 (6023.2- 8529.2) | 95736 | 246.3 (162.5- 345.9) |
|  | 25-29 | 4747138 | 10406.8 (9136.4- 11766.0) | 137440 | 301.3 (193.5- 433.8) |
|  | 30-34 | 8723849 | 13917.2 (12204.2- 15627.7) | 224508 | 358.2 (230.2- 517.5) |
|  | 35-39 | 9635560 | 17723.5 (15943.6- 20028.1) | 220024 | 404.7 (254.9- 612.1) |
|  | 40-44 | 10918882 | 23268.6 (20548.4- 26331.0) | 228214 | 486.3 (298.4- 749.0) |
|  | 45-49 | 18576991 | 33136.8 (29257.1- 37616.0) | 377011 | 672.5 (409.5- 1039.9) |
|  | 50-54 | 27911383 | 45650.8 (40898.6- 51560.8) | 577069 | 943.8 (572.6- 1422.7) |
|  | 55-59 | 33898500 | 61764.4 (54565.2- 70411.9) | 757360 | 1379.9 (842.8- 2154.3) |
|  | 60-64 | 27222143 | 74326.0 (65196.2- 83869.5) | 693131 | 1892.5 (1147.7- 2806.9) |
|  | 65-69 | 31448163 | 83328.3 (73663.7- 89938.3) | 937811 | 2484.9 (1600.2- 3625.2) |
|  | 70-74 | 22800646 | 88181.5 (78238.5- 94141.9) | 794434 | 3072.5 (2066.0- 4302.0) |
|  | 75-79 | 13870058 | 88896.8 (80507.0- 94696.8) | 560893 | 3594.9 (2504.8- 4926.4) |
|  | 80-84 | 7754165 | 89385.5 (81559.8- 95172.9) | 354673 | 4088.5 (2906.7- 5447.9) |
|  | 85-89 | 3114259 | 89466.7 (81252.9- 95122.0) | 157472 | 4523.9 (3295.9- 6035.6) |
|  | 90-94 | 719266 | 88926.2 (81594.8- 95232.3) | 38605 | 4773.0 (3492.6- 6345.4) |
|  | 95+ | 107063 | 88523.4 (78226.3- 95350.8) | 5588 | 4620.6 (3334.8- 6137.7) |

ASR: age-standardized rate; YLDs, years of life lived with disability.

**Supplementary Table 2.** Changes in number of hearing loss prevalence and YLDs according to population-level determinants and causes from 1990 to 2021 in China.

| **Sex** | Overll difference ^a^ | Change due to Population-level determinants (% contribute to the total changes) | | | Overll difference ^e^ | Change due to Population-level determinants (% contribute to the total changes) | | |
| --- | --- | --- | --- | --- | --- | --- | --- | --- |
|  |  | Aging ^b^ | Population ^c^ | Epidemiological change ^d^ |  | Aging^f^ | Population ^g^ | Epidemiological change ^h^ |
| Male | 122923757.7 | 83667948.42(68.06%) | 29391731.74(23.91%) | 9864077.50(8.02%) | 3570655.2 | 2329166.10(65,23%) | 802206.26(22.47%) | 439282.84(12.30%) |
| Female | 124577117.8 | 86039091.66(69.06%) | 29621958.31(23.78%) | 8916067.82(7.16%) | 3556975.1 | 2403282.34(67.57%) | 810442.60(22.78%) | 343250.17(9.65%) |
| Both | 247500875.5 | 169826224.30(68.62%) | 59052704.09(23.86%) | 18621947.02(7.52%) | 7127630.3 | 4732361.47(66.39%) | 1613643.03(22.64%) | 781625.82(10.97%) |

a. Change in prevalence number between year 2021 and 1990

b. Change in prevalence number due to change in the age structure

c. Change in prevalence number due to change in population number

d. Change in prevalence number due to epidemiologic changes. Epidemiologic changes refer to the YLDs number change when age structure and population hold constant

e. Change in YLDs number between year 2021 and 1990

f. Change in YLDs number due to change in the age structure

g. Change in YLDs number due to change in population number

h. Change in YLDs number due to epidemiologic changes. Epidemiologic changes refer to the YLDs number change when age structure and population hold constant

YLDs: Years Lived with Disability

**Supplementary Table** **3.** Join-point regression analysis: trends in age-standardized prevalence and YLDs rates (per 100,000 population) among both sexes, males, and females in China, 1990–2021.

| Sex | Age-standardized prevalence rate | | | Age-standardized YLDs rate | | |
| --- | --- | --- | --- | --- | --- | --- |
|  | Period | APC (95%CI) | AAPC (95%CI) | Period | APC (95%CI) | AAPC (95%CI) |
| Both | 1990-1994 | 0.12 (0.08- 0.16) ^***^ | 0.19(0.18- 0.21) ^***^ | 1990-1998 | -0.09 (-0.10 - -0.07) ^***^ | 0.28 (0.26 - 0.31)  ^***^ |
|  | 1994-2001 | 0.04 (0.02- 0.06) ^***^ |  | 1998-2001 | 0.09 (-0.06 - 0.24) |  |
|  | 2001-2010 | 0.27(0.25-0.28) ^***^ |  | 2001-2004 | 1.21 (1.07 - 1.36) ^***^ |  |
|  | 2010-2015 | 0.21(0.17-0.25) ^***^ |  | 2004-2015 | 0.24 (0.23 - 0.25) ^***^ |  |
|  | 2015-2019 | 0.48(0.42-0.54) ^***^ |  | 2015-2019 | 0.89 (0.82 - 0.96) ^***^ |  |
|  | 2019-2021 | -0.06(-0.18-0.06) |  | 2019-2021 | -0.29 (-0.43 - -0.14) ^***^ |  |
| Female | 1990-2002 | 0.08(0.07-0.08) ^***^ | 0.19 (0.18-0.20) ^***^ | 1990-2000 | 0.03(-0.06-0.12) ^***^ | 0.26 (0.24 - 0.28)  ^***^ |
|  | 2002-2006 | 0.17(0.14-0.20) ^***^ |  | 2000-2005 | -0.04 (-0.06 - -0.03) ^***^ |  |
|  | 2006-2009 | 0.33(0.27-0.39) ^***^ |  | 2005-2015 | 0.65 (0.59 - 0.70) ^***^ |  |
|  | 2009-2015 | 0.27(0.25-0.28) ^***^ |  | 2015-2019 | 0.21 (0.20 - 0.23) ^***^ |  |
|  | 2015-2019 | 0.47(0.44-0.50) ^***^ |  | 2019-2021 | 0.91 (0.82 - 1.00) ^*^ |  |
|  | 2019-2021 | -0.07(-0.13-0.01) ^*^ |  |  |  |  |
| Male | 1990-1995 | 0.13 (0.10- 0.17) ^***^ | 0.20 (0.18 - 0.21) ^***^ | 1990-2001 | -0.08 (-0.10 - -0.07) ^***^ | 0.31 (0.28 - 0.34)  ^***^ |
|  | 1995-2000 | -0.01(-0.06- 0.03) |  | 2001-2004 | 1.68 (1.45 - 1.91) ^***^ |  |
|  | 2000-2009 | 0.31 (0.30- 0.33) ^***^ |  | 2004-2014 | 0.23 (0.21 - 0.25) ^***^ |  |
|  | 2009-2015 | 0.16 (0.13- 0.19) ^***^ |  | 2014-2019 | 0.77 (0.70 - 0.84) ^***^ |  |
|  | 2015-2019 | 0.48 (0.42- 0.54) ^***^ |  | 2019-2021 | -0.28 (-0.50 - -0.06) ^*^ |  |
|  | 2019-2021 | -0.07(-0.20- 0.06) |  |  |  |  |

^*^: 0.01<*P*<0.05; ^**^: 0.001<*P*<0.01; ^***^: *P*<0.001

APC, annual percent change; AAPC, average annual percent change presented for full period; CI, confidence interval; YLDs, years lived with disability.

**Supplementary Table** **4.** Age, period and cohort effects of age-standardized rates of prevalence and YLDs of hearing loss from 1992-2021 in China

| Factors | Prevalence | | | YLDs | | |
| --- | --- | --- | --- | --- | --- | --- |
|  | RR | 95% CI | | RR | 95% CI | |
|  |  | Lower | Upper |  | Lower | Upper |
| Age |  |  |  |  |  |  |
| <5 | 0.856 | 0.841 | 0.871 | 0.851 | 0.831 | 0.871 |
| 5-9 | 0.869 | 0.856 | 0.883 | 0.867 | 0.849 | 0.886 |
| 10-14 | 0.883 | 0.870 | 0.896 | 0.884 | 0.866 | 0.902 |
| 15-19 | 0.897 | 0.885 | 0.909 | 0.901 | 0.884 | 0.917 |
| 20-24 | 0.911 | 0.900 | 0.922 | 0.918 | 0.902 | 0.934 |
| 25-29 | 0.925 | 0.915 | 0.935 | 0.935 | 0.920 | 0.950 |
| 30-34 | 0.939 | 0.930 | 0.949 | 0.953 | 0.939 | 0.967 |
| 35-39 | 0.954 | 0.945 | 0.963 | 0.971 | 0.957 | 0.984 |
| 40-44 | 0.969 | 0.961 | 0.978 | 0.989 | 0.976 | 1.003 |
| 45-49 | 0.984 | 0.976 | 0.993 | 1.008 | 0.995 | 1.021 |
| 50-54 | 1.000 | 0.991 | 1.009 | 1.027 | 1.014 | 1.041 |
| 55-59 | 1.015 | 1.006 | 1.025 | 1.047 | 1.032 | 1.061 |
| 60-64 | 1.031 | 1.021 | 1.042 | 1.066 | 1.051 | 1.082 |
| 65-69 | 1.047 | 1.036 | 1.059 | 1.087 | 1.070 | 1.103 |
| 70-74 | 1.064 | 1.051 | 1.077 | 1.107 | 1.089 | 1.125 |
| 75-79 | 1.080 | 1.066 | 1.095 | 1.128 | 1.108 | 1.148 |
| 80-84 | 1.097 | 1.081 | 1.114 | 1.150 | 1.128 | 1.172 |
| 85-89 | 1.114 | 1.097 | 1.133 | 1.171 | 1.147 | 1.196 |
| 90-94 | 1.132 | 1.112 | 1.152 | 1.194 | 1.167 | 1.221 |
| >95 | 1.150 | 1.128 | 1.172 | 1.216 | 1.187 | 1.246 |
| Period |  |  |  |  |  |  |
| 1996 | 0.976 | 0.970 | 0.983 | 0.968 | 0.958 | 0.978 |
| 2001 | 0.986 | 0.980 | 0.992 | 0.969 | 0.959 | 0.978 |
| 2006 | 1.000 | 1.000 | 1.000 | 1.000 | 1.000 | 1.000 |
| 2011 | 1.018 | 1.012 | 1.024 | 1.016 | 1.007 | 1.025 |
| 2016 | 1.033 | 1.026 | 1.039 | 1.028 | 1.019 | 1.037 |
| 2021 | 1.055 | 1.048 | 1.062 | 1.062 | 1.052 | 1.072 |
| Cohort |  |  |  |  |  |  |
| 1897-1901 | 0.902 | 0.645 | 1.261 | 0.827 | 0.567 | 1.208 |
| 1902-1906 | 0.907 | 0.815 | 1.009 | 0.830 | 0.736 | 0.936 |
| 1907-1911 | 0.914 | 0.873 | 0.958 | 0.836 | 0.792 | 0.882 |
| 1912-1916 | 0.919 | 0.896 | 0.943 | 0.840 | 0.814 | 0.868 |
| 1917-1921 | 0.921 | 0.905 | 0.937 | 0.846 | 0.827 | 0.866 |
| 1927-1931 | 0.924 | 0.912 | 0.936 | 0.857 | 0.841 | 0.873 |
| 1932-1936 | 0.936 | 0.926 | 0.946 | 0.878 | 0.863 | 0.892 |
| 1937-1941 | 0.951 | 0.942 | 0.960 | 0.900 | 0.887 | 0.914 |
| 1942-1946 | 0.961 | 0.953 | 0.969 | 0.921 | 0.909 | 0.934 |
| 1947-1951 | 0.967 | 0.960 | 0.975 | 0.941 | 0.929 | 0.954 |
| 1952-1956 | 0.976 | 0.969 | 0.983 | 0.962 | 0.950 | 0.974 |
| 1957-1961 | 0.987 | 0.980 | 0.994 | 0.981 | 0.969 | 0.993 |
| 1962-1966 | 1.000 | 1.000 | 1.000 | 1.000 | 1.000 | 1.000 |
| 1967-1971 | 1.010 | 1.003 | 1.018 | 1.013 | 1.000 | 1.026 |
| 1972-1976 | 1.012 | 1.004 | 1.020 | 1.014 | 1.000 | 1.028 |
| 1977-1981 | 1.014 | 1.004 | 1.023 | 1.017 | 1.001 | 1.033 |
| 1982-1986 | 1.024 | 1.012 | 1.036 | 1.031 | 1.011 | 1.050 |
| 1987-1991 | 1.044 | 1.030 | 1.058 | 1.054 | 1.032 | 1.076 |
| 1992-1996 | 1.072 | 1.056 | 1.088 | 1.085 | 1.061 | 1.110 |
| 1997-2001 | 1.114 | 1.094 | 1.135 | 1.128 | 1.098 | 1.159 |
| 2002-2006 | 1.178 | 1.148 | 1.209 | 1.172 | 1.133 | 1.213 |
| 2007-2011 | 1.255 | 1.211 | 1.301 | 1.209 | 1.159 | 1.260 |
| 2012-2016 | 1.344 | 1.281 | 1.409 | 1.239 | 1.177 | 1.305 |
| 2017-2021 | 1.413 | 1.320 | 1.513 | 1.257 | 1.170 | 1.350 |

RR, rate ratio; YLDs: Years Lived with Disability

**Supplementary Table** **5.** Predicted trends of prevalence and YLDs of hearing loss in China over the next 15 years (2022–2036)

| Sex | Year | Prevalence | | YLDs | |
| --- | --- | --- | --- | --- | --- |
|  |  | Number | ASR | Number | ASR |
| Both | 2022 | 453626615.6 | 21955.75 | 12662282.7 | 631.44 |
|  | 2023 | 462093546.0 | 21920.71 | 12942845.8 | 633.91 |
|  | 2024 | 470593884.2 | 21914.18 | 13219112.1 | 637.08 |
|  | 2025 | 479192107.3 | 21929.51 | 13495378.5 | 640.07 |
|  | 2026 | 487790330.3 | 21949.12 | 13771644.8 | 642.47 |
|  | 2027 | 496388553.4 | 21958.08 | 14047911.2 | 644.26 |
|  | 2028 | 504986776.5 | 21952.11 | 14324177.6 | 645.68 |
|  | 2029 | 513584999.6 | 21936.98 | 14600443.9 | 647.02 |
|  | 2030 | 522183222.6 | 21922.17 | 14876710.3 | 648.48 |
|  | 2031 | 530781445.7 | 21914.19 | 15152976.6 | 650.13 |
|  | 2032 | 539379668.8 | 21913.61 | 15429243.0 | 651.93 |
|  | 2033 | 547977891.9 | 21916.48 | 15705509.3 | 653.80 |
|  | 2034 | 556576114.9 | 21917.96 | 15981775.7 | 655.66 |
|  | 2035 | 565174338.0 | 21915.47 | 16258042.1 | 657.48 |
|  | 2036 | 573772561.1 | 21909.51 | 16534308.4 | 659.24 |
| Female | 2022 | 221990146.6 | 20982.71 | 6207869.8 | 595.34 |
|  | 2023 | 226689184.5 | 21024.80 | 6371711.9 | 601.13 |
|  | 2024 | 231388222.3 | 21092.95 | 6535554.0 | 606.24 |
|  | 2025 | 236087260.2 | 21154.70 | 6699396.1 | 608.38 |
|  | 2026 | 240786298.0 | 21188.07 | 6863238.2 | 608.22 |
|  | 2027 | 245485335.9 | 21197.17 | 7027080.3 | 608.24 |
|  | 2028 | 250184373.7 | 21204.54 | 7190922.4 | 610.16 |
|  | 2029 | 254883411.6 | 21230.30 | 7354764.5 | 613.60 |
|  | 2030 | 259582449.4 | 21276.70 | 7518606.6 | 616.83 |
|  | 2031 | 264281487.3 | 21329.32 | 7682448.7 | 618.60 |
|  | 2032 | 268980525.1 | 21371.27 | 7846290.7 | 619.17 |
|  | 2033 | 273679563.0 | 21396.92 | 8010132.8 | 619.79 |
|  | 2034 | 278378600.8 | 21414.41 | 8173974.9 | 621.38 |
|  | 2035 | 283077638.7 | 21436.98 | 8337817.0 | 623.80 |
|  | 2036 | 287776676.5 | 21471.61 | 8501659.1 | 626.17 |
| Male | 2022 | 231506936.3 | 22877.25 | 6480258.6 | 666.31 |
|  | 2023 | 234733505.0 | 22786.08 | 6602106.8 | 666.94 |
|  | 2024 | 237960073.7 | 22742.60 | 6723955.0 | 669.18 |
|  | 2025 | 241186642.4 | 22764.56 | 6845803.2 | 672.31 |
|  | 2026 | 244413211.1 | 22844.35 | 6967651.4 | 675.57 |
|  | 2027 | 247639779.8 | 22956.53 | 7089499.6 | 678.41 |
|  | 2028 | 250866348.6 | 23069.93 | 7211347.8 | 680.65 |
|  | 2029 | 254092917.3 | 23159.14 | 7333196.0 | 682.41 |
|  | 2030 | 257319486.0 | 23211.86 | 7455044.1 | 683.90 |
|  | 2031 | 260546054.7 | 23230.45 | 7576892.3 | 685.39 |
|  | 2032 | 263772623.4 | 23228.16 | 7698740.5 | 687.01 |
|  | 2033 | 266999192.1 | 23222.60 | 7820588.7 | 688.83 |
|  | 2034 | 270225760.9 | 23228.98 | 7942436.9 | 690.79 |
|  | 2035 | 273452329.6 | 23255.50 | 8064285.1 | 692.83 |
|  | 2036 | 276678898.3 | 23302.00 | 8186133.3 | 694.86 |

ASR: age-standardized rate (per100,000 population), YLDs: Years Lived with Disability

**Supplementary Table** **6.** The results of stationarity tests and white noise tests of the ARIMA model

| Metrics | Sex | Final Select Model  (p, d, q) | KPSS test | | Ljung-Box test | |
| --- | --- | --- | --- | --- | --- | --- |
|  |  |  | KPSS Level | *P* | χ^2^ | *P* |
| Number |  |  |  |  |  |  |
| Prevalence | Male | (0,2,2) | 0.183 | >0.1 | 2.127 | 0.713 |
|  | Female | (0,2,2) | 0.136 | >0.1 | 3.775 | 0.437 |
|  | Both | (0,2,4) | 0.164 | >0.1 | 1.036 | 0.904 |
| YLDs | Male | (0,2,1) | 0.087 | >0.1 | 7.773 | 0.100 |
|  | Female | (0,2,1) | 0.072 | >0.1 | 7.432 | 0.191 |
|  | Both | (0,2,3) | 0.082 | >0.1 | 3.831 | 0.429 |
| ASR |  |  |  |  |  |  |
| Prevalence | Male | (2,1,1) | 0.100 | >0.1 | 0.337 | 0.997 |
|  | Female | (2,2,0) | 0.132 | >0.1 | 2.649 | 0.754 |
|  | Both | (2,2,0) | 0.139 | >0.1 | 0.859 | 0.973 |
| YLDs | Male | (2,1,1) | 0.129 | >0.1 | 3.970 | 0.554 |
|  | Female | (3,1,1) | 0.190 | >0.1 | 2.777 | 0.734 |
|  | Both | (2,1,1) | 0.211 | >0.1 | 3.023 | 0.6964 |

ASR: age-standardized rate (per100,000 population), YLDs: Years Lived with Disability

**Supplementary Table** **7.** The specific parameters of the final ARIMA model

| Metrics | Sex | Final Select Model  (p, d, q) | AIC | BIC | ME | RMSE | MAE | MPE | MAPE | MASE | ACF1 |
| --- | --- | --- | --- | --- | --- | --- | --- | --- | --- | --- | --- |
| Number |  |  |  |  |  |  |  |  |  |  |  |
| Prevalence | Male | (0,2,2) | 829.35 | 833.55 | -661.55 | 207814.90 | 169205.70 | 0.00 | 0.10 | 0.04 | 0.10 |
|  | Female | (0,2,2) | 823.42 | 827.62 | 22598.44 | 180897.10 | 147285.00 | 0.02 | 0.10 | 0.04 | -0.19 |
|  | Both | (0,2,4) | 863.66 | 870.67 | 40347.10 | 334536.00 | 250247.60 | 0.02 | 0.08 | 0.03 | -0.04 |
| YLDs | Male | (0,2,1) | 653.37 | 656.17 | 700.30 | 11463.73 | 8072.66 | 0.02 | 0.18 | 0.07 | -0.13 |
|  | Female | (0,2,1) | 654.93 | 657.74 | 2177.20 | 12038.33 | 7724.72 | 0.06 | 0.17 | 0.07 | -0.02 |
|  | Both | (0,2,3) | 691.56 | 697.17 | 2060.69 | 19592.85 | 13624.41 | 0.03 | 0.15 | 0.06 | 0.07 |
| ASR |  |  |  |  |  |  |  |  |  |  |  |
| Prevalence | Male | (2,1,1) | 261.29 | 268.46 | 0.64 | 13.28 | 9.48 | 0.00 | 0.04 | 0.20 | 0.04 |
|  | Female | (2,2,0) | 225.33 | 229.53 | -0.23 | 9.96 | 6.55 | 0.00 | 0.03 | 0.16 | 0.05 |
|  | Both | (2,2,0) | 237.47 | 241.67 | -1.17 | 11.92 | 8.05 | -0.01 | 0.04 | 0.19 | 0.03 |
| YLDs | Male | (2,1,1) | 93.26 | 100.43 | 0.03 | 0.85 | 0.59 | 0.00 | 0.09 | 0.23 | 0.03 |
|  | Female | (3,1,1) | 83.79 | 92.40 | 0.07 | 0.71 | 0.50 | 0.01 | 0.09 | 0.29 | 0.01 |
|  | Both | (2,1,1) | 86.65 | 93.82 | 0.03 | 0.77 | 0.57 | 0.00 | 0.10 | 0.27 | 0.05 |

ASR: age-standardized rate (per100,000 population), YLDs: Years Lived with Disability

**Supplementary Table** **8.** Test for number of Joinpoints

| Test For Number of Joinpoints | | | | | | | | | |
| --- | --- | --- | --- | --- | --- | --- | --- | --- | --- |
| Sex | Test number | Null Hypothesis | Alt Hypothesis | Selected Hypothesis | Numerator DF | Denominator DF | Number of Permutations | P-Value | Significance level^*^ |
| Both | 0 | 0 | 5 | 5 | 10 | 20 | 4500 | 0.0002 | 0.0100 |
| Both | 1 | 1 | 5 | 5 | 8 | 20 | 4500 | 0.0002 | 0.0125 |
| Both | 2 | 2 | 5 | 5 | 6 | 20 | 4500 | 0.0002 | 0.0167 |
| Both | 3 | 3 | 5 | 5 | 4 | 20 | 4500 | 0.0007 | 0.0250 |
| Both | 4 | 4 | 5 | 5 | 2 | 20 | 4500 | 0.0011 | 0.0500 |
| Female | 0 | 0 | 5 | 5 | 10 | 20 | 4500 | 0.0002 | 0.0100 |
| Female | 1 | 1 | 5 | 5 | 8 | 20 | 4500 | 0.0002 | 0.0125 |
| Female | 2 | 2 | 5 | 5 | 6 | 20 | 4500 | 0.0002 | 0.0167 |
| Female | 3 | 3 | 5 | 5 | 4 | 20 | 4500 | 0.0007 | 0.0250 |
| Female | 4 | 4 | 5 | 5 | 2 | 20 | 4500 | 0.0113 | 0.0500 |
| Male | 0 | 0 | 5 | 5 | 10 | 20 | 4500 | 0.0002 | 0.0100 |
| Male | 1 | 1 | 5 | 5 | 8 | 20 | 4500 | 0.0002 | 0.0125 |
| Male | 2 | 2 | 5 | 5 | 6 | 20 | 4500 | 0.0002 | 0.0167 |
| Male | 3 | 3 | 5 | 5 | 4 | 20 | 4500 | 0.0002 | 0.0250 |
| Male | 4 | 4 | 5 | 5 | 2 | 20 | 4500 | 0.0007 | 0.0500 |

*: Significance level for individual test; Final Select Model: Both - 5 joinpoints; Female - 5 joinpoints; Male - 5 joinpoints
